# In vitro inactivation of *Mycobacterium avium* subsp. *paratuberculosis* (MAP) by use of copper ions

**DOI:** 10.1186/s12866-018-1313-6

**Published:** 2018-11-01

**Authors:** P. Steuer, C. Avilez, C. Tejeda, N. Gonzalez, A. Ramirez-Reveco, F. Ulloa, A. Mella, I. R. Grant, M. T. Collins, M. Salgado

**Affiliations:** 1 Instituto de Medicina Preventiva Veterinaria, Facultad de Ciencias Veterinarias, Universidad Austral de Chile, Saelzer Building 5° Floor, Campus Isla Teja, PO Box 567, Valdivia, Chile; 2 Instituto de Ciencia Animal, Universidad Austral de Chile, Valdivia, Chile; 3 Escuela de Graduados, Facultad de Ciencias Veterinarias, Universidad Austral de Chile, Valdivia, Chile; 4 Instituto de Bioquímica y Microbiología, Universidad Austral de Chile, Valdivia, Chile; 5 Institute for Global Food Security, School of Biological Sciences, Queen’s University Belfast, Belfast, Northern Ireland UK; 6 Department of Pathobiological Sciences, School of Veterinary Medicine, University of Wisconsin, Madison, USA

**Keywords:** *Mycobacterium avium* subsp. *paratuberculosis*, Copper ions, Phosphate buffered saline, MGIT culture, Phage amplification assay

## Abstract

**Background:**

*Mycobacterium avium* subsp. *paratuberculosis* (MAP) is the causative agent of paratuberculosis, a contagious infectious disease that affects domestic and wild ruminants causing chronic inflammation of the intestine. MAP has proven to be very resistant to both physical and chemical processes, making it difficult to control this pathogen. Based on the recognized antimicrobial properties of copper, the objective of this study was to evaluate the effectiveness of copper ions to reduce MAP numbers and/or MAP viability in a fluid matrix. Besides, methicillin-resistant *Staphylococcus aureus* (MRSA), and *Escherichia coli* were used as controls of the effectiveness of copper ions. MAP-spiked PBS was subjected to copper ions treatment at 24 V for 5 min and the PBS suspensions were sampled before and after treatment. MAP viability and quantification were determined using three complementary techniques: a phage amplification assay, MGIT culture and qPCR.

**Results:**

Moderate numbers (10^3^ CFU ml^−1^) of the two control bacteria were completely eliminated by treatment with copper ions. For MAP, copper ions treatment reduced both the viability and numbers of this pathogen. Phage assay information quickly showed that copper ions (24 V for 5 min) resulted in a significant reduction in viable MAP. MGIT culture results over time showed statistically significant differences in time-to-detection (TTD) values between PRE and POST treatment. MAP genome equivalent estimates for PBS suspensions indicated that MAP numbers were lower in samples POST-treatment with copper ions than PRE-treatment.

**Conclusions:**

The use of copper ions resulted in a significant reduction of MAP in a liquid matrix, although some MAP survival on some occasions was observed.

## Background

*Mycobacterium avium* subsp. *paratuberculosis* (MAP) is the causative agent of a highly contagious infectious disease known as paratuberculosis or Johne’s disease. It affects mainly domestic and wild ruminants, but also affects a wide range of non-ruminant animal species [1, 2], including humans [3]. MAP is one of the most fastidious members of the *Mycobacterium* genus and belongs to the *Mycobacterium avium* complex (MAC). It is a Gram-positive bacterium that is acid-fast due to its thick cell wall rich in complex lipids [2]. It has an extremely low metabolic activity and tends to form clumps or “clusters” of bacterial cells [2]. In addition, it can form biofilms [4]. These biological features make MAP highly resistant to adverse environmental conditions, such as low pH, high salt concentrations, and chlorine [5], and allows its survival for more than a year in the environment [6]. In addition, this pathogen has shown resistance to high temperatures, e.g. multiple studies show that although several logs of MAP are killed, a number of MAP cells are able to survive pasteurization [7, 8]. Similarly, MAP has been recovered from retail high-temperature short-time (HTST) pasteurized milk (72 °C for 15–25 s) [9]. Moreover, viable MAP has been recovered from powdered infant formula [10] and in calf milk replacer [11]. Because of the importance of MAP for both animal and public health, there is an urgent need to find better methods to destroy the organism and thereby limit transmission to animals and humans.

Numerous studies have confirmed the antimicrobial properties of copper and copper alloys. Copper surfaces can eliminate nosocomial infectious agents such as *Staphylococcus aureus, Enterobacter aerogenes,* Methicillin-resistant *Staphylococcus aureus* (MRSA)*,* and *Pseudomonas aeruginosa, Escherichia coli* O157:H7 and other bacterial pathogens, viruses and fungi [12–16]. Interestingly, Mehtar et al. [17] reported significant inactivation of two clinical isolates of *Mycobacterium tuberculosis* after exposure to copper surfaces.

The use of copper as an antimicrobial was officially approved for use in 2008 by the United States Environmental Protection Agency [18]. The aim of the present study was to evaluate the ability of copper ions to reduce viable MAP counts.

## Results

As controls, the effectiveness of copper ions to inactivate, MRSA and *E. coli* cells suspended in PBS was assessed. Suspensions of both MRSA and *E. coli* (≥ 3 log_10_ CFU mL^−1^) were completely inactivated by copper ions; no colonies were observed on blood agar post-treatment.

**Fig. 1:**
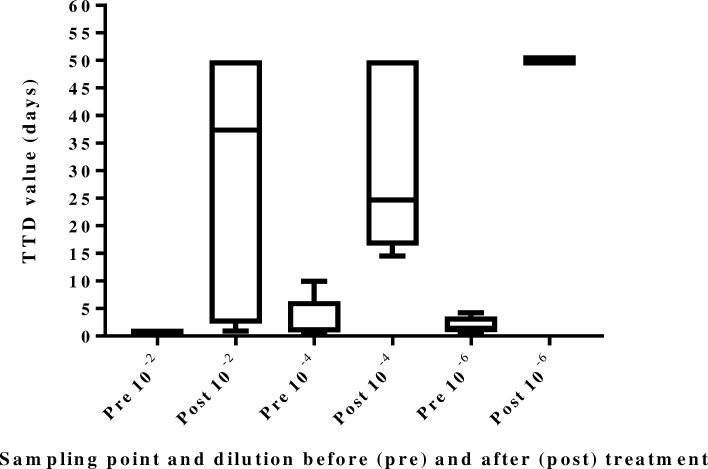
Relationship between MAP load in three different PBS dilutions before (PRE) and after (POST) treatment with copper ions and MGIT culture time to detection (TTD) values, expressed in days; the higher the TTD value the lower the number of viable MAP present

To evaluate the effect of copper ions on MAP viability, both a phage amplification assay and Mycobacterial Growth Indicator Tube (MGIT) culture followed by quantitative Polymerase Chain Reaction (qPCR) confirmation (culture-PCR) were used. Phage assay results showed that copper ions (24 V for 5 min) resulted in a significant reduction (*P* = 0.03) in viable MAP for all MAP dilutions (Table 1). MGIT culture results showed statistically significant differences in time-to-detection (TTD) values between PRE and POST treatment (*P* < 0.002 for 10^−2^ and 10^−4^ MAP dilution and *P* < 0.0001 for the 10^−6^ MAP dilution (Fig. 1).

**Table 1:** Summary of phage assay counts (PFU mL^−1^) for three dilutions of MAP-spiked PBS before (PRE) and after (POST) treatment with copper ions at 24 V for 5 min; indicating significant reductions (*P* = 0.03) in numbers of viable MAP

Replicate experiment	MAP PBS dilution^a^:					
	10^−2^		10^−4^		10^−6^	
	PRE	POST	PRE	POST	PRE	POST
I	5.8 × 10^8^	3.0 × 10^7^	4.5 × 10^6^	< 1	9.1 × 10^4^	< 1
II	4.6 × 10^8^	2.0 × 10^7^	3.0 × 10^6^	< 1	6.3 × 10^4^	4.0 × 10^3^
III	5.6 × 10^8^	< 1	3.3 × 10^6^	< 1	5.3 × 10^4^	< 1
IV	3.5 × 10^8^	< 1	3.1 × 10^6^	< 1	5.0 × 10^4^	< 1
V	3.0 × 10^8^	< 1	3.0 × 10^6^	< 1	3.0 × 10^4^	< 1
VI	7.1 × 10^8^	2.0 × 10^7^	3.0 × 10^6^	< 1	3.0 × 10^4^	< 1
Mean PFU mL^−1^ ± SD	4.9 × 10^8^ ± 1.5 × 10^8^	1.2 × 10^7^ ± 1.3 × 10^7^	3.3 × 10^6^ ± 5.9 × 10^5^	< 1	5.3 × 10^4^ ± 2.3 × 10^4^	6.7 × 10^2^ ± 16 × 10^2^

^a^Only experimental results validated with proper positive and negative control results have been included in this table

The genome equivalent principle method for MAP count estimation was adopted [19]. MAP genome equivalent estimates for Phosphate Buffer Saline (PBS) suspensions indicated that MAP numbers were lower in samples POST-treatment with copper ions than PRE- treatment for all dilutions tested (Table 2).

**Table 2:** Estimated mean MAP load determined by qPCR at different dilutions (10^−2^, 10^−4^ and 10^−6^) before (PRE) and after (POST) treatment with copper ions, for each replicate experiment. Data represent mean number of MAP DNA copies (genome equivalents) per 200 μL of PBS, from which DNA was extracted

Replicate experiment	MAP dilution:					
	10^−2^		10^−4^		10^−6^	
	PRE	POST	PRE	POST	PRE	POST
I	1.9 × 10^6^	1.4 × 10^3^	1.5 × 10^3^	3.2 × 10^2^	1.3 × 10^2^	ND^a^
II	2.8 × 10^6^	ND	5.5 × 10^2^	ND	4.7 × 10^1^	ND
III	9.4 × 10^5^	1.2 × 10^1^	9.2 × 10^2^	1.6 × 10^1^	3.3 × 10^2^	ND
IV	1.9 × 10^5^	1.1 × 10^1^	1.5 × 10^3^	1.2 × 10^1^	2.7 × 10^2^	ND
V	1.6 × 10^5^	3.2 × 10^2^	3.3 × 10^3^	2.1 × 10^1^	3.1 × 10^3^	ND

^a^ND, no MAP DNA detected by qPCR

**Fig. 2:**
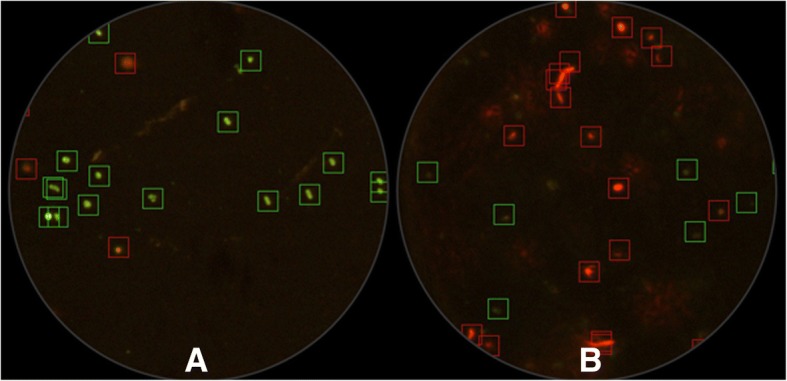
Representative fields captured by epifluorescent microscopy during plasma membrane integrity analysis; green and red fluorescent marks correspond to cells recognized as live and dead, respectively. The images (60X magnification) correspond to a PBS suspension containing 10^6^ cells mL^−1^ stained with LIVE/DEAD BacLight Bacterial Viability kit before (**a**) and after (**b**) exposure to copper ions

Examination of LIVE/DEAD BacLight™ stained samples at 1000 magnification (oil immersion) showed that copper ions reduced the percentage of viable MAP when compared to the pre-treatment control. In addition, live cells in the post-treatment samples showed altered cell conformation and lower fluorescence emission (Fig. 2). The vast majority of copper ion-treated MAP cells stained with SYTO9 and propidium iodide emitted red fluorescence, indicating that their cell membranes were clearly affected, and allowing uptake of propidium iodide into the cell.

## Discussion

The complete inactivation of the control bacteria (*E. coli* and MRSA) by copper ions in this study was consistent with prior reports demonstrating that copper surfaces inactivated up to 99.9% of these bacteria [13–15]. We first tested the copper device without electric current stimulation of the copper plates and we observed also a total killing effect for the control bacteria as when the electric current was applied to the copper plates. Meanwhile, for MAP a deleterious effect of copper ions treatment on cell viability was also observed, but in a longer time than when the electric current was used to stimulate the copper plates (data not shown). Therefore, it may be concluded that the latter helps to release more copper ions from the metal matrix to have a greater killing effect.

Regarding the methods of viable MAP detection applied during this study, the phage assay allowed us to quickly assess (within 24 h) the immediate effect of copper ions, and MGIT culture results subsequently provided evidence of recovery from adverse effects of copper ions over time, i.e. possible MAP tolerance and resilience to copper ions. Staining of MAP cells with BacLight™ stain provided information about possible damage to cell membrane structures, and the qPCR assay provided information regarding MAP DNA integrity post treatment. The four methods were applied in parallel during this study to provide us with a full picture about the effects of copper ions on the viability of this important animal pathogen and the potential mode of action of the copper ions treatment.

It has been established that TTD values are correlated with MAP CFU counts [20]. However, as some MAP concentrations were outside the optimal range for conversion of TTD to CFU mL^−1^, we used the raw TTD data for analysis.

Since PCR cannot normally distinguish between viable and dead cells, the expectation would have been that MAP counts estimated by qPCR should have been similar pre- and post- copper ions treatment. However, we found significantly lower MAP numbers in post-treatment samples, and qPCR negative results were quite common; which may be evidence that copper ions damage MAP DNA making it non-amplifiable, as has been reported previously for other pathogens [21, 22]. This observation agrees with the study of Espiritu Santo et al. [22] using *E. coli* and other bacteria, where they concluded that copper acts mainly by damaging the cell envelope. However, it differs from a study using MRSA in which copper had little effect on membrane integrity and its antimicrobial effect may have been due to cell respiratory disruption and/or genomic DNA damage [23]. Although there is no published literature to date explaining the potential antimicrobial effect of copper ions on MAP, exposure to copper surfaces was detrimental to *M. tuberculosis* viability [17]. This antimicrobial activity may be associated with one or more possible mechanisms, e.g. altering membrane integrity, damaging the microbial DNA, and altering protein synthesis [12, 16, 21]. Our qPCR results suggest that copper ions cause DNA damage in MAP cells.

The observed MAP copper tolerance may be due to the tendency of MAP cells to cluster thereby protecting cells in the center, as proposed by Rowe et al. [24] regarding MAP resistance to heat. The lipid-rich, hydrophobic cell wall of MAP likely makes MAP innately more resistant to copper ions than MRSA and *E. coli*. Additionally, some MAP cells may have survived exposure to copper ions because they were present in a non-replicating, inactive, dormant, spore-like state [25, 26].

## Conclusions

In summary, this study has demonstrated for the first time that copper ions have a substantial killing effect on MAP in vitro when suspended in PBS, although MAP survival on some occasions was observed. Whether such treatment represents an effective and practical decontamination tool for MAP, and also elucidating the exact mechanism of action of copper ions treatment, will be the subject of future research.

## Material and methods

### Bacterial strains and inoculum preparation

The study organisms were MAP (American Type Culture Collection (ATCC) 19,698), methicillin-resistant *Staphylococcus aureus* (ATCC 43300), and *Escherichia coli* (ATCC 25922). *S. aureus* and *E. coli* were included as controls to confirm copper treatment efficacy on Gram-positive and Gram-negative bacteria [12–15]. MRSA and *E. coli* strains were kept at −80 °C, then cultured on blood agar plates (Oxoid Diagnostic Reagents, UK) containing 5% lamb’s blood incubated at 37 °C for 24 h. One colony was transferred to a tube of Brain Heart Infusion (Oxoid Diagnostic Reagents, UK) and incubated overnight at 37 °C on an orbital shaker. The broth was then centrifuged at 3000 x *g* for 15 min at 4 °C, and the pellet was resuspended in 10 mL of sterile PBS. Bacterial concentrations were determined using serial 10-fold dilutions in PBS plate counting on blood agar. For testing, the concentration of bacteria was adjusted using PBS to obtain a final concentration of 1000 CFU mL^−1^.

MAP was cultured in 40 mL 7H9 broth supplemented with 10% oleic acid-albumin-dextrose- catalase (OADC) (Becton Dickinson and Company, USA), 2 mg mL^−1^ Mycobactin J (Allied Monitor, USA) and 5 mL L^−1^ glycerol for 1 month at 37 °C. MAP cultures were declumped by vortexing with sterile 3 mm glass beads. MAP growth was monitored weekly using a Helios Gamma1 spectrophotometer (Thermo Scientific). When the absorbance at 600 nm reached a value of 1.0, it was estimated be in late exponential growth at a concentration of ~ 10^8^ MAP cells mL^−1^ with minimal dead cells present [20]. Ten-fold serial dilutions of MAP were made in PBS and 10^−2^, 10^−4^ and 10^−6^ dilutions were used for copper inactivation experiments. All bacterial suspensions were kept at 4 °C for no longer than 24 h until use.

### Copper treatment

**Fig. 3:**
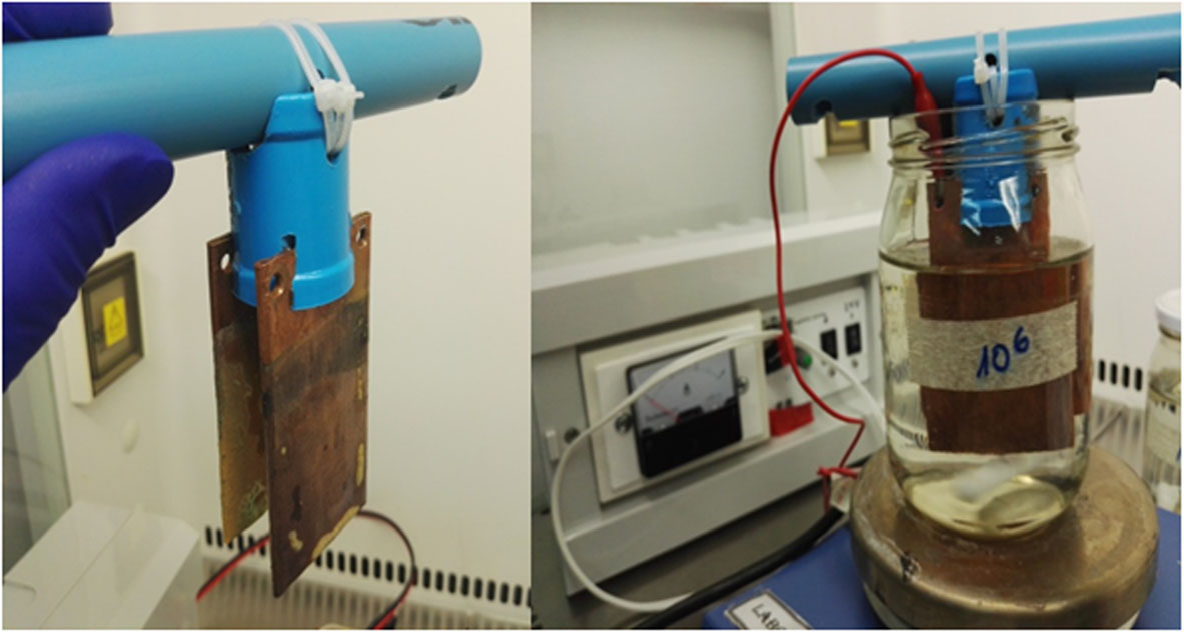
Laboratory apparatus used for treatment of bacterial cell suspensions with copper ions, consisting of a glass receptacle within which copper plates of high purity were immersed in PBS. The copper plates were stimulated with a low voltage (24 V) electric current to generate copper ions, and suspensions were mixed by means of a magnetic stirrer during treatment

A copper treatment device consisting of a glass receptacle containing 300 mL PBS (0.5X) in which two high purity copper plates were immersed was used. The copper plates were stimulated with a low voltage (24 V) electric current (3 Amperes) to quickly release large concentrations of copper ions. A magnetic stirrer placed in the glass receptacle allowed constant mixing during treatment (Fig. 3).

### Test protocol

Copper-treated and non-treated suspensions of MAP (10^6^ CFU mL^−1^, 10^4^ CFU mL^−1^, 10^2^ CFU mL^−1^), and MRSA and *E. coli*, both at 10^3^ CFU mL^−1^ were tested. The negative control was unspiked PBS not treated with copper ions. Each treatment was independently replicated five times in duplicate. Bacterial suspensions were sampled before and after each copper treatment.

### Evaluation of MAP, MRSA and *E. coli* viability and enumeration

MAP viability and quantification was determined using three complementary techniques: a phage amplification assay, MGIT culture and qPCR. The phage-based method exploits the ability of D29 mycobacteriophage to replicate within and subsequently lyse only viable cells of mycobacteria. Products of phage amplification following infection are observed as lysed areas (plaques), which can be recorded after 24 h on indicator plates prepared with fast-growing *Mycobacterium smegmatis* [27]. The phage amplification assay was used to obtain a rapid estimation (within 24–48 h) of MAP numbers and viability as described by Foddai and Grant [28] with minor modifications. Hundred μL of MAP PBS suspension were added to 900 mL of 7H9/OADC/CaCl_2_ broth (i.e. 10^−1^ dilution), incubated overnight at 37 °C, and used as sample to be tested by the phage assay. The number of plaque forming units (PFU) has been correlated with the number of colony forming units (CFU) of MAP using the phage amplification assay (r^2^ = 0.9514) previously [28]. To confirm that plaques were due to MAP, up to 10 plaques were cut from the agar and pooled, before DNA was extracted and tested to confirm MAP by a previously published real-time IS*900* PCR method [29]. Primer sequences, which amplified a 63-nucleotide fragment of the IS*900* gene target, were 5′-GACGCGATGATCGAGGAG-3′ and 5′-GGGCATGCTCAGGATGAT-3′, and the probe sequence was 5′ 6-FAM/ACCTCCGTAACCGTCATTGTCCAGATCA/3′ BHQ-1. In parallel with the phage amplification assay, 100 μL of MAP PBS suspensions were inoculated into the liquid culture BACTEC-MGIT 960 system (Becton Dickinson, Sparks, MD), used according to manufacturer’s instructions, in order to evaluate the ability of MAP to recover viability or repair damage and grow after treatment. Furthermore, BACTEC-MGIT 960 culture system allowed us to make a semi-quantitative assessment of MAP load by determining the time taken for culture tubes to signal positive (time to detection, TTD). To each MGIT tube was added 800 μL of MGIT ParaTB supplement (Becton Dickinson, Sparks, MD), and 500 μL of egg yolk suspension (Becton Dickinson, Sparks, MD). In order to avoid antibacterial effect other than copper ions, no polymyxin B, amphotericin B, nalidixic acid, trimethoprim, and azlocillin (PANTA) antimicrobial cocktail was added to MGIT tubes. A 200 μl aliquot of all growth positive MGIT cultures was subjected to DNA extraction and purification according to a published protocol [6], then confirmed molecularly to be MAP positive by a real-time PCR technique [6].

Bacterial load (genome equivalent) from MAP PBS suspensions, before and after treatment, were estimated, according to a published protocol [19], based on the concentration of MAP DNA that was measured in a Nanoquant spectrophotometer (TECAN group, Männedorf, Schweiz) adjusted for a 10^8^ dilution and the number of copies of the IS*900* target gene, and having the reference of the molecular weight of the genome of MAP ATCC strain 19,698 to establish a standard curve for estimation of MAP numbers in the sample by Roche 2.0 real-time PCR, according to the following equation:

$$ Genome\ equivalent=\frac{DNA\  concentration\ \left( ng/\mu l\right)\times \left(6.022\times {10}^{23}{mol}^{-1}\right)}{\begin{array}{c}\left(4.829\times {10}^6 base\ pairs\right)\times \left(1\times {10}^9 ng/g\times 660g/ mol\right)\\ {}\left( MAP\  ATCC\ 19698\  genome\right)\ \left( Base\ mass\right)\end{array}} $$

For MRSA and *E. coli* strains, bacterial concentrations in each sample were determined using serial 10-fold dilutions plated on blood agar. From each plate, a colony typical of *S. aureus* was identified using Staphytec Plus (Oxoid Diagnostic Reagents, UK) [30], or a colony typical of *E. coli* was identified using a panel of biochemical tests, which included Triple sugar iron agar, Lysine iron agar, Motility indole ornithine medium, Simmon’s citrate agar and Urea agar (all from Oxoid Diagnostic Reagents, UK).

To complement assessment of MAP viability by the phage amplification assay and MGIT culture, a Live/Dead staining technique (Live/Dead *Bac*Light bacterial viability kit, Invitrogen) was applied to PBS suspensions, according to the manufacturer’s instructions, to differentiate cells treated with or without copper ions with undamaged and damaged permeable membranes. Stained samples were analysed using the computer-assisted sperm analysis (CASA) system (viability module of the Sperm Class Analyser®, Microptic, Spain), previously calibrated to bacterial size (field), and coupled to an epifluorescence microscope (Nikon E200, Japan) with a high-velocity camera (Basler AG, Germany). Viability percentages were established from a minimum of 200 cells for each sample obtained in different fields.

### Statistical analysis

To test the assumption of normality of the obtained results, Shapiro-Wilk’s test was used. The statistical significance of the increase or reduction in plaque counts (PFU mL^−1^), MAP counts estimation (genome equivalent), and TTD observed for each treatment applied to buffer samples inoculated with viable MAP was assessed by Wilcoxon signed rank test (not normally distributed data). All data analyses were performed in GraphPad Prism 7.04 and differences with *P* < 0.05 were considered significant.

## Abbreviations

ATCC: American Type Culture Collection
CASA: Computer-assisted sperm analysis
CFU: Colony forming units
HTST: High-temperature short-time
MAC: *Mycobacterium avium* complex
MAP: *Mycobacterium avium* subsp. *paratuberculosis*
MGIT: Mycobacterial growth indicator tube
MRSA: methicillin-resistant *Staphylococcus aureus*
OADC: Oleic acid-albumin-dextrose- catalase
PANTA: Polymyxin B, amphotericin B, nalidixic acid, trimethoprim, and azlocillin
PBS: Phosphate saline buffer
PFU: Plaque forming units
qPCR: Quantitative polymerase chain reaction
TTD: Time to detection
